# Whey Protein Hydrolysate Increases Translocation of GLUT-4 to the Plasma Membrane Independent of Insulin in Wistar Rats

**DOI:** 10.1371/journal.pone.0071134

**Published:** 2013-08-30

**Authors:** Priscila Neder Morato, Pablo Christiano Barboza Lollo, Carolina Soares Moura, Thiago Martins Batista, Rafael Ludemann Camargo, Everardo Magalhães Carneiro, Jaime Amaya-Farfan

**Affiliations:** 1 University of Campinas (UNICAMP), Faculty of Food Engineering (FEA), Campinas, São Paulo, Brazil; 2 University of Campinas (UNICAMP), Institute of Biology (IB), Campinas, São Paulo, Brazil; Universidad Miguel Hernández de Elche, Spain

## Abstract

Whey protein (WP) and whey protein hydrolysate (WPH) have the recognized capacity to increase glycogen stores. The objective of this study was to verify if consuming WP and WPH could also increase the concentration of the glucose transporters GLUT-1 and GLUT-4 in the plasma membrane (PM) of the muscle cells of sedentary and exercised animals. Forty-eight Wistar rats were divided into 6 groups (n = 8 per group), were treated and fed with experimental diets for 9 days as follows: a) control casein (CAS); b) WP; c) WPH; d) CAS exercised; e) WP exercised; and f) WPH exercised. After the experimental period, the animals were sacrificed, muscle GLUT-1 and GLUT-4, p85, Akt and phosphorylated Akt were analyzed by western blotting, and the glycogen, blood amino acids, insulin levels and biochemical health indicators were analyzed using standard methods. Consumption of WPH significantly increased the concentrations of GLUT-4 in the PM and glycogen, whereas the GLUT-1 and insulin levels and the health indicators showed no alterations. The physical exercise associated with consumption of WPH had favorable effects on glucose transport into muscle. These results should encourage new studies dealing with the potential of both WP and WPH for the treatment or prevention of type II diabetes, a disease in which there is reduced translocation of GLUT-4 to the plasma membrane.

## Introduction

Different food proteins may affect muscle metabolism in different fashions, as shown when the effects of whey proteins (WP) are compared to those of casein (CAS) [1]–[3]. One difference between WP and CAS is that WP stimulates an increase of fatty acid synthesis in the muscle accompanied by a concomitant decrease in fatty acid synthesis in the liver, considered to be a positive effect on lipid metabolism [4]. Hydrolysis of the protein alone could alter the biological function of the protein, thus affecting the metabolism [5]. For example, it has been suggested that a slight change in the physicochemical form of the protein when presented to the animal can be enough to influence the general metabolism, apparently as a result of the various peptides that are generated during partial enzymatic hydrolysis of whey proteins (WPH) [3], [6]–[8]. The bioactive peptides present in WPH might be capable of reducing the levels of creatine kinase in soccer players [9].

Alterations in glycogen metabolism have most likely been the most frequently reported positive feature resulting from substituting WP with WPH [7], [8], [10]. Morifuji et al. [6] showed that from seven possible dipeptides containing the branched-chain amino acids found in milk-whey proteins, the peptide Ile-Leu was capable of increasing the uptake of glucose by isolated rat muscle. These reports suggest that WP promotes physiological responses different from those of CAS, such as greater endurance exercise performance [11], better post-exhaustion glycemic levels and higher glycogen levels [7], [8], [10]. Muscle glycogen is the primary fuel source during prolonged moderate-to-high intensity exercise [12], so an increase in glycogen could mean increased physical performance.

Two isoforms from the facilitative glucose transporter family, GLUT-4 and GLUT-1, are expressed in skeletal muscle [13]. GLUT-1 is present in very low amounts in skeletal muscle and has been suggested to influence basal glucose uptake by the muscle [14]. The abundance of GLUT-4 protein is a primary factor in determining the maximal rate of glucose transport into skeletal muscle. Under normal resting conditions, most of the GLUT-4 molecules reside in membrane vesicles inside the muscle cell. In response to insulin or muscle contractions, GLUT-4 translocates to the cell membrane, where it is inserted to increase glucose transport [15], [16].

Given that consumption of either whey protein (WP) or whey protein hydrolysate (WPH) results in an increase in glycogen stores in skeletal muscle, the present study was designed to investigate the hypothesis that such an effect could be due to increased translocation of GLUT-4 to the plasma membrane. For this purpose, several parameters of metabolism were determined that included the following: the levels of GLUT-4, GLUT-1, p85, Akt, phosphorylated-Akt, glycogen, serum insulin, and plasma amino acids in Wistar rats. These parameters were assessed for the two types of protein, using casein as control, under two states of physical activity, sedentary and exercised.

## Materials and Methods

**Figure 1:**
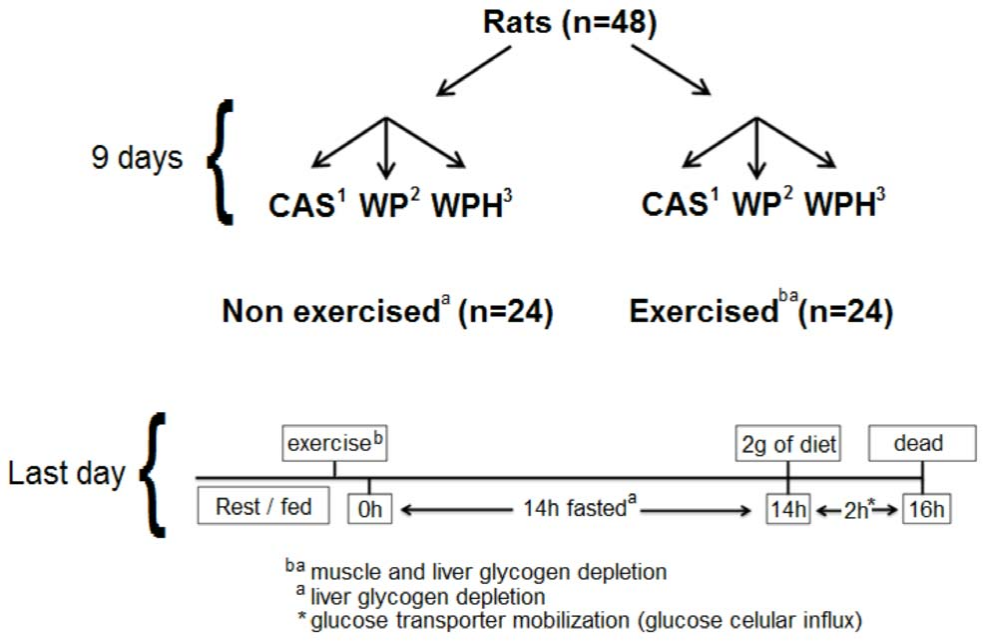
Experimental design. Forty-eight Wistar rats were divided into 6 groups (n = 8 per group), corresponding to the three diets: casein (CAS), whey protein (WP), whey protein hydrolysate (WPH) and two exercise regimes (sedentary and exercise). The animals fed with experimental diets for 9 days, and the last day, all of the rats had fasted overnight to arrive at similar glycogen reserves, but two hours before sacrifice, they each received 2 g of the appropriate experimental diet.

Forty-eight male Wistar rats (∼150 g; n = 8 per group) were divided into sedentary and exercised groups, and each group was fed casein (CAS, control), whey protein (WP) or whey protein hydrolysate (WPH) as their dietary protein source for a total of 9 days. All the animals were fasted overnight to arrive at similar glycogen reserves, but two hours before sacrifice, each received 2 g of the appropriate experimental diet (Figure 1). Food consumption was determined every other day, and body mass was monitored weekly. When the animals reached ∼150 g, they were randomly assigned to groups and body mass gain was checked after one week. The research methodology was approved by the Ethics Committee on Animal Experimentation (CEEA-UNICAMP, protocol 2376-1/2011).

The molecular weight distribution of the WPH peptides showed 40.5% <1 kDa, 26.7% between 1 and 5 kDa, and 15.6% between 5 and 20 kDa.

### Training Protocol

Exercised rats underwent training on a treadmill, running for 60 minutes at 15 m/min 16 h before sacrifice. A single exercise session enhanced GLUT-4 translocation to the plasma membrane [15], [16]. This procedure allowed the analyses to be carried out with the animals homogeneously depleted of glycogen and at a moment of high glucose influx into their cells, 16 h after exercise, when the expression of GLUT-4 was maximum [17].

### Experimental Diets

The diets (Table 1) were isonitrogenous (approximately 12% protein, dry weight basis), isolipidic and isocaloric (approximately 360 kcal/100 g), and each was formulated following the recommendations of the American Institute of Nutrition, AIN93-G diet [18]. The three diets differed from each other with respect to the nature of the protein source; the control diet was casein, and the two experimental diets were whey protein diets, one of which was pre-hydrolysed, (Hilmar product 8350) and the other was not (Hilmar product 8000). The complete amino acid profile of each protein was determined (Table 2). The degree of hydrolysis in the pre-hydrolysed whey protein was approximately 12.5%, and both whey proteins were donated by Hilmar Ingredients (Dalhart, TX, USA). The standard reference protein was casein.

**Table 1:** Composition of diets (g/kg of diet).

Ingredient	CAS (g)	WP (g)	WPH (g)
Corn Starch	437.92	427.31	425.00
Dextrinized starch	145.42	141.90	141.13
Sucrose	110.16	107.50	106.92
WPH	–	–	156.41
WP	–	152.77	–
CAS	135.96	–	–
Vegetable oil	70.00	70.00	70.00
Fiber (cellulose)	50.00	50.00	50.00
Mineral mixture	35.00	35.00	35.00
Vitamin mixture	10.00	10.00	10.00
L-Cystine	3.00	3.00	3.00
Choline bitartrate	2.50	2.50	2.50
Tert-butylhydroquinone	0.014	0.014	0.014

CAS: casein, WP: whey protein, WPH: whey protein hydrolysate.

Density caloric: approximately 360 kcal/100 g.

**Table 2:** Amino acid profile of the protein sources (g/100 g of protein), dry basis.

Amino Acid	CAS	SEM	WP	SEM	WPH	SEM
Asparagine	5.93	0.02	11.50	0.03	11.14	0.03
Glutamate	18.98	0.1	18.80	0.1	17.97	0.3
Serine	4.69	0.02	5.30	0.02	5.06	0.02
Glycine	1.40	0.01	1.70	0.01	1.77	0.01
Histidine	2.12	0.01	1.30	0.01	1.29	0.02
Arginine	3.01	0.01	2.64	0.01	2.33	0.02
Threonine	3.58	0.01	7.62	0.01	7.45	0.03
Alanine	2.32	0.01	5.10	0.02	4.87	0.02
Proline	8.83	0.03	5.90	0.03	5.66	0.02
Tyrosine	4.57	0.02	2.85	0.02	2.77	0.02
**Methionine**	**2.32**	0.02	**2.51**	0.01	**2.52**	0.02
**Cystine**	**0.16**	0.00	**1.48**	0.01	**1.60**	0.01
Isoleucine	4.50	0.01	6.87	0.02	6.95	0.02
Leucine	7.64	0.02	10.10	0.02	10.13	0.03
Valine	5.36	0.02	5.66	0.03	5.80	0.02
Phenylalanine	3.9	0.03	2.84	0.02	2.79	0.02
Lysine	6.60	0.02	9.21	0.02	9.47	0.02
**MET+CYST** a	**2.48**		**3.99**		**4.12**	

a Sum of methionine plus cysteine.

### Biochemical Parameters

Blood samples were collected in Vacutainers, maintained at 4°C and then centrifuged at 3000 *g* (4°C, 12 min) to obtain the serum. The assessment of the sera included the following: uric acid, urea, aspartate aminotransferase (AST), alanine aminotransferase ALT, creatine kinase (CK) and lactate dehydrogenase (LDH). The standard enzymatic spectrophotometric determinations were carried out employing Laborlab kits (São Paulo, Brazil). For the glycogen analysis, samples of the skeletal muscle, heart and liver were collected. Tissue glycogen was isolated and purified by precipitation with ethanol after basic digestion, and then it was quantified by the phenol-sulfuric acid method [19]. The serum insulin levels were measured using a rat/mouse insulin ELISA (Millipore), and the glucose concentrations were measured using an Accu-Chek Active glucometer (Roche Diagnostics, Mannheim, Germany).

### Protein Extraction and Immunoblotting

For the determination of GLUT-4 and GLUT-1 in the plasma membrane (PM), the tissue samples (skeletal muscle) were homogenized in buffer (10 mM Tris HCl, 1 mM EDTA, and 250 mM sucrose) and subjected to differential centrifugations [20]. The total protein content of the skeletal muscle was determined by the Lowry method [21]. For immunoblotting, the tissue (∼100 mg) homogenates were subjected to SDS-PAGE and transferred to a nitrocellulose membrane using the Wide Biocom Western blot system (Bridge of Weir, UK). The blots were probed with the appropriate antibodies against GLUT-4 (Abcam, Cambridge; catalogue number ab654, diluted 1∶5000), GLUT-1 (Abcam, Cambridge; catalogue number ab40084, diluted 1∶1000), Akt 1/2/3 (H-136) antibody SC8312 (Santa Cruz Biotechnology, CA, USA diluted 1∶1000), p-Akt 1,2,3 Ser 473, antibody SC7985-R (Santa Cruz Biotechnology, CA, USA diluted 1∶1000), and Anti-PI 3-Kinase p85, N-SH2 domain (catalogue number #06-496, Upstate Biotechnology NY, USA diluted 1∶1000). To assess the levels of these proteins in the muscle, the loading control was tubulin (Abcam, Cambridge, catalogue number ab44928, diluted 1∶1000) and anti-Na^+^K^+^Cl^-^ - Co-transporter 2 (Millipore, catalogue number AB2281, diluted 1∶1000), the appropriate secondary antibody conjugated to peroxidase and the BM chemiluminiscence blotting system were used for detection. The bands were visualised by chemiluminiscence (GE – ImageQuant LAS4000, Piscataway, NJ, USA), and the band intensities were quantified by scanning and processed using the ImageJ program (v. 1.44 for Windows).

### Determination of Free Amino Acids in the Plasma

The free amino acids were extracted from the plasma using methanol and derivatized with phenylisothiocyanate [22], and the PTH-derivatives were chromatographed using a Luna C-18, 100 Å; 5 µ, 250×4.6 mm (00 G-4252-EQ) column at 50°C. Quantification was performed by comparison with a standard mixture, and the internal standard was DL-2-aminobutyric acid (Sigma-Aldrich Corp, St Louis, MO, USA). The free amino acids were extracted in 80% ethanol and 0.1 M HCl, with 500 µL of 2-aminobutyric acid added as the internal standard. The mixture was sonicated for 10 min and further homogenized for 1 h, followed by centrifugation at 8,500 *g* for 15 min. The supernatant was filtered through a 0.22 mm membrane, a 40 µL aliquot was derivatized as described above, and 20 µL was injected into the liquid chromatograph.

### Statistical Analysis

The results were subjected to statistical analysis using the software SPSS (Statistical Package for the Social Sciences, Chicago, United States), version 17.0. The data were tested for normality (Kolmogorov-Smirnov test) and homogeneity using the tools available therein. For the parametric data, the multivariate analysis of variance (ANOVA) was used, and the means were compared (Duncan test), adopting the value of *p*<0.05 as the criterion for statistical significance.

## Results

**Figure 2:**
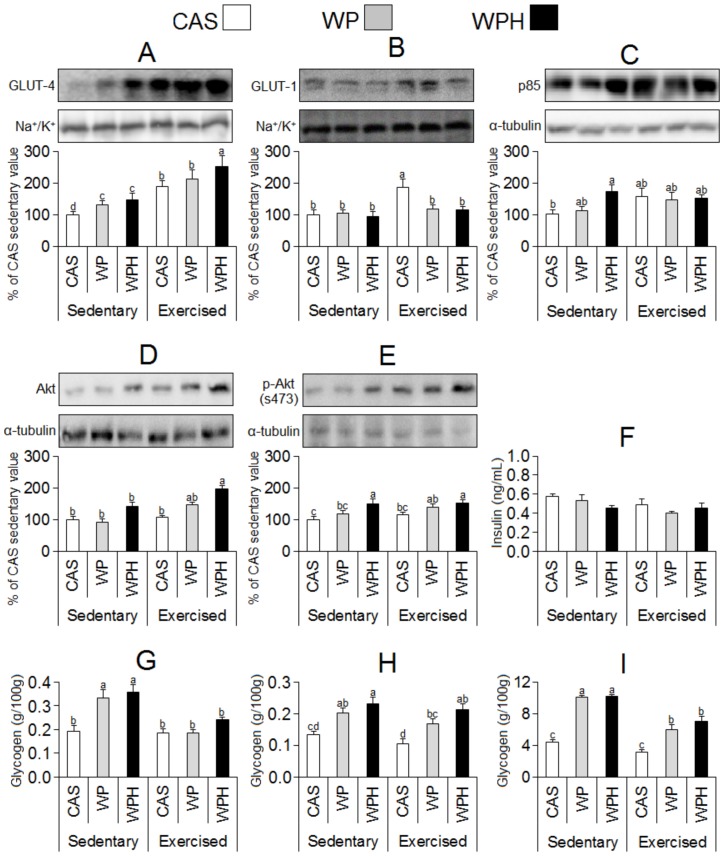
Effect of different dietary proteins in glucose homeostasis. Means and standard error for the concentrations of: **A)** GLUT-4; **B)** GLUT-1; **C)** p85/tubulin; **D)** Akt/tubulin; **E)** Akt phosphorylated (Ser 473)/tubulin in the skeletal muscle; **F)** insulin in serum; and of glycogen : **G)** heart; **H)** skeletal muscle and **I)** liver. The effect of 9 days of dietary whey protein (WP [gray bar], n = 8) and whey protein hydrolysate (WPH [black bar], n = 8) on sedentary and exercised (in treadmill) Wistar rats. The control group received the standard casein diet (CAS [white bar], n = 8). The AIN93-G WP and WPH diets were prepared by substituting casein of the standard AIN93 diet with whey protein or whey protein hydrolysate. ANOVA was used for statistical treatment of the data, and the means were compared (Duncan test), adopting the value of *p*<0.05 as the criterion for statistical significance. Different superscript lowercase letters indicate significant differences between the groups.

The consumption of whey protein (WP) and whey protein hydrolysate (WPH) for 9 days resulted in a significant increase (*p*<0.05) in GLUT-4 translocation to the plasma membrane (Figure 2A). It was evident that exercise had a magnifying effect on translocation for all food proteins, but particularly for the whey proteins. Phosphorylation of Akt at serine 473 (Figure 2E) was increased (*p*<0.05) by the consumption of whey protein hydrolysate in both groups, sedentary and exercised, whereas there was no difference in the serum insulin concentrations between the control group (CAS, with casein as dietary protein) and the WPH group. However, the consumption of WPH also increased glycogen concentrations in the heart, skeletal muscle and liver (Figure 2G–I).

The following general health blood parameters were accessed: glucose, uric acid and urea. In addition, AST and ALT were determined as hepatic health parameters, and CK and LDH were assessed as muscle damage indicators (Table 3). No differences (*p*>0.05) were observed.

**Table 3:** Biochemical blood parameters in serum.

	Sedentary						Exercised					
	CAS		WP		WPH		CAS		WP		WPH	
	Mean	SEM	Mean	SEM	Mean	SEM	Mean	SEM	Mean	SEM	Mean	SEM
LDH	778.92	67.89	954.02	34.43	947.48	25.82	855.64	58.57	940.01	30.45	949.75	36.58
CK	951.95	136.81	942.63	107.98	1262.58	132.24	1343.93	119.60	1104.57	105.31	1192.15	123.65
ALT	24.14	2.1	24.36	2.41	27.69	1.49	24.13	2.22	27.45	2.60	34.19	3.83
AST	114.64	11.19	128.12	3.21	145.18	7.43	126.32	7.50	134.23	9.59	120.79	17.06
HEMO	17.43^a^	0.83	18.67^a^	0.65	17.21^a^	0.33	13.61^b^	0.55	13.25^b^	0.61	14.55^b^	0.67
TPROT	5.80^ab^	0.36	5.83^ab^	0.26	6.49^a^	0.10	5.51^ab^	0.22	4.92^b^	0.28	5.39^b^	0.18
Urea	22.22^c^	1.86	22.19^c^	1.39	22.96^bc^	1.48	30.17^a^	0.80	27.61^ab^	1.29	27.37^ab^	1.95
UA	0.70	0.06	0.77	0.05	0.88	0.07	0.87	0.07	0.85	0.04	0.70	0.04
Glucose	137.13	3.70	142.25	4.30	140.38	4.13	141.88	5.70	135.13	3.50	140.00	3.05

LDH: lactate dehydrogenase (U/L); CK: creatine kinase (U/L); ALT: alanine aminotransferase (U/L); AST: aspartate aminotransferase (U/L); HEMO: hemoglobin (mg/DL); TPROT: total proteins (mg/DL); mg/DL; UA: uric acid (mg/DL); mg/DL. The effect of 9 days of dietary whey protein (WP) and whey protein hydrolysate (WPH) on the serum homeostasis parameters of sedentary and exercised (in treadmill) Wistar rats, the control group receiving the standard diet with casein (CAS, n = 8). The AIN93-G WP and WPH diet was made by substituting the casein (standard diet) for whey protein (WP, n = 8) or whey protein hydrolysate (WPH, n = 8). Different superscript lowercase letters indicate significant differences between the groups. ANOVA was used and the means compared (Duncan test), adopting the value of *p*<0.05 as the criterion for statistical significance.

The change in dietary protein had no effect (*p*<0.05) on growth, the weight of the organs, the organ weight/body weight ratio (data not shown) or the biochemical indicators of health and renal and liver function (Table 3). The complete amino acid profiles (Table 4) were obtained, and of the alterations detected, the most notable in the context of this study was the decrease in the plasmatic levels of taurine in the exercised animals.

**Table 4:** Mean (M) and standard error of the mean (SEM) of amino acid concentrations in plasma.

	Sedentary						Exercised					
	CAS		WP		WPH		CAS		WP		WPH	
	Mean	SEM	Mean	SEM	Mean	SEM	Mean	SEM	Mean	SEM	Mean	SEM
ASP (3.04)	59,43^b^	3,45	64,02^ab^	2,56	66,39^a^	2,19	65,54^a^	3,21	67,05^a^	3,69	65,39^a^	2,42
GLU (3.37)	68,70	2,13	57,20	2,46	27,08	0,84	29,45	1,00	20,36	1,14	15,68	0,63
HPRO (4.62)	52,62	2,68	50,10	1,75	43,00	1,46	49,15	2,51	48,87	2,93	52,43	2,25
ASN (5.89)	65,60	2,62	61,41	2,40	63,01	1,95	68,99	4,07	63,50	2,86	66,44	3,65
SER (6.19)	158,30^a^	5,54	125,27^ab^	4,26	134,44^ab^	5,51	168,11^a^	6,72	151,27^a^	6,96	104,40^b^	5,74
GLN (6.61)	661,50^a^	27,12	651,40^a^	38,43	703,86^a^	28,15	554,14^b^	26,60	569,75^b^	20,51	557,25^b^	20,06
GLY (6.87)	174,35	9,24	165,37	5,46	287,97	15,26	218,08	9,16	164,57	8,23	284,61	10,25
HIS (8.00)	13,86^b^	0,69	24,79^a^	0,92	25,90^a^	0,85	16,31^a^	0,77	19,67^a^	0,75	15,50^a^	0,56
**TAU (9.34)**	**168,83^ab^**	**6,25**	**183,32^a^**	**10,27**	**163,70^ab^**	**8,35**	**111,47^c^**	**3,34**	**155,39^ab^**	**6,22**	**137,25^b^**	**4,94**
ARG (9.80)	21,43^ab^	1,26	10,15^b^	0,57	18,47^b^	0,70	43,21^a^	1,51	11,06^b^	0,65	3,31b	0,11
THR (10.85)	148,93^c^	6,26	246,99^ab^	12,60	249,29^ab^	9,97	198,34^bc^	8,13	284,09^a^	9,66	198,86^bc^	11,14
ALA (11.70)	478,76	17,24	507,19	18,77	551,80	33,11	519,43	25,97	551,27	24,26	515,67	29,39
PRO (13.35)	174,18^a^	6,62	85,09^b^	4,94	100,82^b^	4,23	218,39^a^	9,61	108,33^b^	3,57	62,08^b^	2,17
TYR (33.53)	40,50^d^	2,23	65,05^bc^	3,12	60,70^c^	3,16	59,48^c^	2,26	84,16^a^	3,20	76,02^ab^	3,27
VAL (37.27)	56,54^a^	3,11	28,82^b^	1,35	51,11^a^	3,07	53,98^a^	2,86	14,00^b^	0,63	16,76^b^	1,01
**MET (40.61)**	**99,28^b^**	**4,17**	**80,01^b^**	**4,32**	**190,78^a^**	**7,82**	**176,31^a^**	**7,40**	**164,69^a^**	**5,93**	**118,82^b^**	**6,89**
**CYS (48.09)**	**62,39**	**2,68**	**62,82**	**2,95**	**62,60**	**2,38**	**59,76**	**2,27**	**66,74**	**2,40**	**73,63**	**2,87**
ILEU (49.19)	30,31^cd^	1,49	32,03^bc^	1,86	21,15^d^	0,72	30,78^bc^	1,02	47,72^a^	1,96	39,83^ab^	1,55
LEU (49.89)	9,47^b^	3,20	18,71^b^	1,07	43,94^a^	1,85	13,66^b^	0,57	7,18b	0,35	13,37^b^	0,67
PHE (56.03)	20,87^b^	0,94	32,65^a^	1,47	15,87^b^	0,94	13,26^b^	0,76	32,24^a^	1,13	20,83^b^	0,79
TRP (59.00)	55,66^b^	2,28	104,04^a^	5,41	79,69^ab^	2,79	69,24^ab^	3,39	60,48^ab^	3,27	94,59^ab^	5,20
LYS (61.12)	466,60	20,06	410,89	18,49	459,01	17,44	440,04	22,00	511,41	27,62	421,97	13,92
Sum	3082,56	114,05	3067,32	159,50	3420,59	194,97	3177,11	168,39	3203,81	169,80	2954,67	177,28

ASP: aspartate, GLU: glutamate, HPRO: hydroxyproline, ASN: asparagine, SER: serine, GLN: glutamine, GLY: glycine, HIS: histidine, ARG: arginine, TAU: taurine, THR: threonine, ALA: alanine, PRO: proline, TYR: tyrosin, VAL: valine, MET: methionine, CYS: cysteine, ILE: isoleucine, LEU: leucine, PHE: phenylalanine, TRP: tryptophan, LYS: lysine. Taurine and their precursors (MET and CYS) amino acids are bolded. The AIN93-G WP and WPH diet was made by substituting the casein (CAS, n = 8) for whey protein (WP, n = 8) or whey protein hydrolysate (WPH, n = 8). Different superscript letters indicate significant differences between groups, ANOVA was used and means were compared (Duncan test), adopting the value of *p*<0.05 as a criterion for statistical significance. Number in parenthesis following each amino acid is the corresponding retention time in minutes.

## Discussion

Based on previous findings by the present [3], [7], [8] and other [10], [23] authors clearly showing that the consumption of WP and WPH raised muscle and hepatic glycogen levels, the objective of the present study was to verify the effect that the consumption of WP and WPH had on the translocation of the glucose transporters GLUT-4 and GLUT-1 to the plasma membrane (PM), as compared to rats fed a standard diet (AIN93-G) with casein as the protein source. The results showed clearly that the consumption of WP and WPH increased the translocation of GLUT-4 (Figure 2A) when compared to the casein-fed animals, whereas GLUT-1 (Figure 2B) was not responsive to the different proteins. This increase in GLUT-4 in the PM was consistent with increases in glycogen (Figure 2G–I) because with more glucose transporters in the cell PM, the availability of glucose and synthesis of glycogen could both increase. Physical exercise is known to increase the potential for the translocation of GLUT-4 to the membrane [16], [24], and for all diets, the exercised animals demonstrated higher levels of GLUT-4 in the PM.

One of the primary means to increase the concentration of GLUT-4 in the plasma membrane is through insulin-regulated trafficking [25]. However, in the present experiment, no increase was noted in serum insulin levels in the groups consuming WPH (Figure 2F). The experimental design of the study focused on the moment of greatest mobilization of glucose transporter-4, and the animals were sacrificed 2 h after consuming the meal; this was too long an interval to observe the maximum plasma insulin response. Previous studies have shown the maximum insulin response is between 20 and 60 min after the ingestion of whey protein [26], [27]. The results obtained for the proteins involved in the insulin signaling route suggested that the total Akt (Figure 2D) as well as the form phosphorylated on serine 473 (Figure 2E) increased significantly (*p*<0.05) in the groups consuming WP and WPH, with the greatest increase occurring in the WPH group. Enhanced phosphorylation of Akt is capable of increasing the mobilization of GLUT-4 to the PM. In contrast, p85, which is also involved in insulin-regulated trafficking, was apparently not affected by consumption of WP or WPH (Figure 2C). Translocation of GLUT-4 to the PM can also be stimulated in an insulin-independent manner. Carneiro et al. [28] accomplished this through taurine activation of the insulin pathway, thus raising the GLUT-4 concentration in the plasma membrane independent of insulin. However, the molecular mechanism behind this effect has still not been elucidated [29]. In the exercised animals of the WP and WPH groups, the plasma concentrations of taurine (Table 4) were greater (p<0.05) than those in the control group consuming CAS. This could explain, at least in part, the greater translocation of GLUT-4 in the WP and WPH groups. After investigating the amino acid composition of the WP and WPH, it was found they were rich in sulfur amino acids (Table 2), and methionine and cysteine are endogenous precursors of taurine [28]. Thus, the consumption of WP or WPH provided a greater amount of substrate for the endogenous production of taurine than casein, and the presence of this amino acid may have facilitated activation of the insulin pathway and cell capture of glucose, as indicated in the literature.

In addition, to explain the results for GLUT-4 (Figure 2A), it is notable that WPH contains peptides with the ability to increase glucose capture and glycogen synthesis in vitro [6]. Morifuji et al. [6] tested the capacity of cells to capture glucose when incubated with 7 different peptides present in WPH and observed that the peptide isoleucyl-leucine significantly increased the uptake of glucose and also the synthesis of glycogen. Morifuji et al. [4] also reported that animals consuming WPH presented a greater capacity to synthesize intramuscular fatty acids, which could be the result of the influx of substrate into the cell for the synthesis of fatty acids. These are effects of WPH, which could be related to the peptides present in this protein, as it has already been reported that milk proteins are rich sources of these substances, which have an important, but frequently unexplained, metabolic role [5].

There was no difference between the groups (Table 3) with respect to the muscle damage markers CK and LDH [30], although soccer players supplemented for 8 weeks with WPH have shown a significant decrease in these markers [9], which normally tend to increase after exercise. In addition, chronic supplementation caused no significant alterations liver (in the analyzed ALT and AST) health parameters. These results are consistent with previous studies showing that the consumption of whey proteins induces no changes in these liver enzymes [1], [2]. With respect to the blood parameters analyzed, hemoglobin, total protein and urea, no differences were observed in relation to the diet, though some alterations could have been induced by exercise. The growth and anthropometric parameters did not suggest alterations in body and organ weight due to the difference in dietary proteins. Taken together, the biochemical and anthropometric parameters suggested that WP and WPH are generally safe for consumption by Wistar rats with respect to the health parameters analyzed in this study.

### Conclusion

The consumption of WP and WPH was capable of increasing translocation of GLUT-4 to the plasma membrane and the glycogen concentration, but did not trigger alterations in insulin levels. The effect was significantly higher in the WPH group, and even greater increases were observed when the animals performed aerobic exercise. These results should encourage further studies considering the potential of WP and WPH in the treatment or prevention of insulin resistance and type 2 diabetes, a disease in which translocation of GLUT-4 to the plasma membrane is reduced.
